# Detection of multi-resistant clinical strains of *E. coli* with Raman spectroscopy

**DOI:** 10.1007/s00216-021-03800-y

**Published:** 2022-01-04

**Authors:** Amir Nakar, Aikaterini Pistiki, Oleg Ryabchykov, Thomas Bocklitz, Petra Rösch, Jürgen Popp

**Affiliations:** 1 Leibniz Institute of Photonic Technology Jena (a Member of Leibniz Health Technologies), Albert-Einstein-Straße 9, 07745 Jena, Germany; 2 Institute of Physical Chemistry and Abbe Center of Photonics, Friedrich Schiller University, Helmholtzweg 4, 07743 Jena, Germany; 3 Research Campus Infectognostics Jena E.V, Philosophenweg 7, 07743 Jena, Germany; 4 Jena Biophotonics and Imaging Laboratory, Albert-Einstein-Straße 9, 07745 Jena, Germany

**Keywords:** Antibiotic resistance, Raman spectroscopy, Bacteria, Label-free, Diagnostic, Machine learning

## Abstract

In recent years, we have seen a steady rise in the prevalence of antibiotic-resistant bacteria. This creates many challenges in treating patients who carry these infections, as well as stopping and preventing outbreaks. Identifying these resistant bacteria is critical for treatment decisions and epidemiological studies. However, current methods for identification of resistance either require long cultivation steps or expensive reagents. Raman spectroscopy has been shown in the past to enable the rapid identification of bacterial strains from single cells and cultures. In this study, Raman spectroscopy was applied for the differentiation of resistant and sensitive strains of *Escherichia coli*. Our focus was on clinical multi-resistant (extended-spectrum β-lactam and carbapenem-resistant) bacteria from hospital patients. The spectra were collected using both UV resonance Raman spectroscopy in bulk and single-cell Raman microspectroscopy, without exposure to antibiotics. We found resistant strains have a higher nucleic acid/protein ratio, and used the spectra to train a machine learning model that differentiates resistant and sensitive strains. In addition, we applied a majority of voting system to both improve the accuracy of our models and make them more applicable for a clinical setting. This method could allow rapid and accurate identification of antibiotic resistant bacteria, and thus improve public health.

**Graphical abstract:**

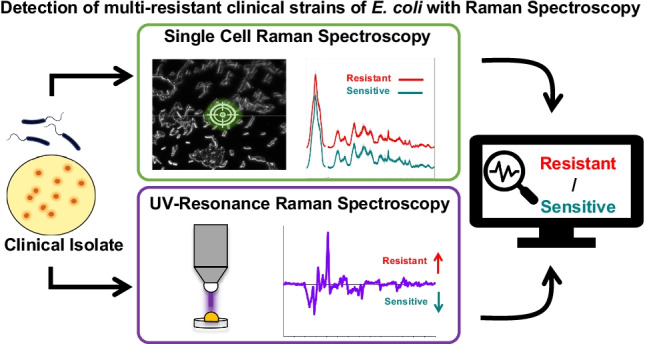

**Supplementary Information:**

The online version contains supplementary material available at 10.1007/s00216-021-03800-y.

## Introduction

The rise of antimicrobial resistance is a global public health challenge [1]. The Organisation for Economic Co-operation and Development (OECD) predicts that 2.4 million people in Europe, North America, and Australia will die from infections caused by resistant microorganisms before 2050, leading to healthcare costs up to US$3.5 billion per year [2, 3]. In a recent study, it was shown that ~ 28% of all resistance-attributed-deaths are caused by *Escherichia coli* strains that are resistant to extended-spectrum β-lactams (ESBLs) or carbapenems (CREs) [2, 4]. As these bacteria are spreading and evolving throughout the world, global actions are taken to reduce the unnecessary use of last-resort antibiotics [1, 3]. However, due to the limitations of microbiological diagnostic methods, this is often not feasible.

Prescribing the correct antibiotic treatment to patients, in time, is of paramount importance for this cause [5]. The routine microbiological techniques used in clinical laboratories require at least 48 h and up to 4 days to deliver results on pathogen resistance [6, 7]. This leads physicians to use empirical treatment based on patient history and resistance rates of healthcare facilities. These treatments are not always appropriate and contribute to further increase antimicrobial resistance, since often last-line antibiotics are used unnecessarily [5, 8].

Therefore, there is a need for rapid and reliable diagnostic procedures to facilitate prompt and effective treatment. Over the past decades, novel molecular diagnostic techniques for the classification of pathogens and detection of resistance have been developed and applied. These methods have improved clinical diagnostics significantly, but have several disadvantages, primarily the high cost of consumables and the need for extensive infrastructure and specially trained personnel [6]. Furthermore, in case of the PCR assays, the method does not apply when new mutations naturally appear as previous knowledge is needed to design the assay [9, 10]. Thus, the need for a sensitive, specific, rapid, and reliable method for antibiotic resistance detection remains.

Raman spectroscopy is a novel technique with high potential for clinical applications. It is rapiand divergent evolution. Thed and label-free and can be applied on bacterial cells with minimal sample preparation [11–13]. Raman spectroscopy probes the chemical fingerprint of bacterial cells and cultures, without the need for external reagents and expensive consumables. Since different strains of bacteria differ in their chemical composition, these differences are captured in their Raman spectrum and can be evaluated using chemometric methods [12, 14].

This technique has been shown in many studies to enable differentiation of bacterial strains and species [14–25]. For example, Raman microspectroscopy of single bacterial cells was used to differentiate strains of *Mycobacteria* [17], *Burkholderia* [16, 26], *Legionella* [19], *Escherichia* [15], and other clinically relevant bacteria [11, 27]. Alternatively, instead of measuring the spectra of single cells, one can also measure colonies or dried biomass. Raman spectroscopy on bulk samples was used to differentiate strains of *Staphylococci* [21, 23, 25], *Pseudomonas* [24], and other clinically relevant bacteria [20, 22].

Bulk measurement has advantages and disadvantages over single-cell analysis: it requires longer cultivation or enrichment, which takes time, but often produces better signal-to-noise ratios (S/N). This improvement is because the signal is coming from thousands of cells at a time. Moreover, in single-cell microspectroscopy, it is not practical to use ultraviolet (UV) illumination as the cell is destroyed from the high energy of the beam. UV light causes photothermal damage from the accumulated thermal energy applied to the sample, causing complex molecular structures to break down and burn. For bulk samples, this problem is mediated by rotating the slide containing the bacterial biomass in a spiral manner during measurement, minimizing local thermal build up. However, in single-cell analysis, the beam must be focused on the cell, destroying it entirely via photothermal damage.

Using UV light is important as it causes a resonance Raman signal enhancement of nucleic acids and aromatic amino acids—which improves S/N in spectra of cells tremendously [28–30]. When using UVRR spectroscopy on microbiological samples, the signals collected are primarily those that are resonance-enhanced—i.e., proteins, DNA, and RNA. On the other hand, if the samples are illuminated with 532 nm light, a more comprehensive look into the biochemistry of the cell is observed, with spectral features of lipids, carbohydrates proteins, and nucleic acids combined. This trade-off between higher S/N ratio and the number of spectral features observable often makes the choice of method for analysis difficult, yet, for difficult classification tasks, UVRR spectroscopy is often preferred [28]. While both single-cell analysis and bulk measurement have advantages and disadvantages important in adjusting Raman-based diagnostics to healthcare, no study so far has compared these two approaches for these applications.

Antibiotic-resistant bacteria have also been analyzed with Raman spectroscopy. For example, by exposing cells to antibiotics, resistant bacteria displayed an increase in a protein marker band and a decrease in a nucleic acid marker [31–33]. Some studies focused on differentiation between strains of antibiotic-resistant bacteria without antibiotic exposure. Namely, methicillin-resistant *Staphylococcus aureus* [23] and multi-resistant *E. coli* [22, 23] were typed using Raman spectroscopy on bulk samples. In those studies, it was concluded that Raman typing can be used to determine whether different clinical isolates are of the same clonal line. Despite the fact that these studies could distinguish the different strains, their approach leads to a purely taxonomic evaluation that is not suitable for everyday clinical laboratory practice. This limits the application of the method to the strains used in the study and does not consider the natural differences that define their resistances. The differences between resistant strains are many and complex, coming from diverse origins and divergent evolution. The resistance genes carried by a strain are, among others, part of the supra genome that contains plasmids, mobile genetic elements, and the bacterial chromosome. This supra genome significantly varies from strain to strain [34–38]. These variations are present even when the strains evolve in similar environments, such as a hospital. Another approach was to use artificially engineered antibiotic-resistant *E. coli* [39]. However, artificial selection for antimicrobial resistance creates strains with less diversity than those which evolve naturally and independently in natural and clinical environments [38].

The aim of the present study was to differentiate clinical multi-resistant (ESBL and CRE) *E. coli* isolates from sensitive strains without exposure to antibiotics. Furthermore, for the first time, the use of Raman microspectroscopy on single cells and UV resonance Raman spectroscopy (UVRR) on bulk samples was compared for the purpose of differentiation between multi-resistant and sensitive bacteria. These findings could be applied to reduce the time, cost, and effort needed to detect antibiotic resistance in clinical settings.

## Material and methods

### Antibiotic susceptibility test

Antibiotic susceptibility to extended-spectrum β-lactam and carbapenem antibiotics was evaluated using a VITEK 2 Compact (bioMérieux) with VITEK® 2 AST cards (bioMérieux). The test was run according to the manufacturer’s instructions. Furthermore, for isolates defined as “pathogenic/sensitive” a minimum inhibitory concentration (MIC) test was conducted for the following antibiotics: tetracycline, streptomycin, polymyxin B, gentamycin, nitrofurantoin, chloramphenicol, trimethoprim, cefuroxime, cefaclor, cefazolin, and amikacin. The tests were conducted using the E-Test (Liofilchem) according to the manufacturer’s instruction. Briefly, for each strain, 0.5 McFarland was spread on Mueller Hinton agar (Merck, Darmstadt, Germany) plates and exposed to a strip of varying antibiotic concentrations. The plates were incubated for 24 h in 37 °C, and the zone of growth inhibition was assessed visually to determine the MIC.

### Sample preparation

The resistance class and sources of all *E. coli* used isolates are shown in Table S1. A total of 20 isolates were used, consisting of 10 multi-resistant (5 ESBL and 5 CRE) and 10 sensitive *E. coli* isolates. On every measurement date, bacteria were transferred from – 80 °C storage to a nutrient agar (NA) plate (Carl Roth) and incubated overnight at 37 °C. A loopful of biomass was transferred from the agar plate to Nutrient Broth media (Carl Roth) and incubated at 37 °C with shaking of 120 rpm. For each isolate, three independent replicates were prepared and measured on different days.

For UVRR measurements, the bacteria were harvested after 1 h of growth in 20 mL of Nutrient Broth media for heat inactivation. Three separate tubes of 1.5 mL inoculum were heat-inactivated at 99 °C for 5 min. The heat-inactivated bacteria were washed 3 times with 1 mL distilled water. After centrifugation at 5000* g* for 5 min (Rotina 380R, Hettich), the supernatant was discarded and the pellet re-suspended in 30 μL of distilled water. Finally, bacteria were loaded onto a fused-silica slide and air dried at room temperature for 1 h. Heat inactivation was verified by plating the bacteria on NA agar plates and observing no colonies after incubation of 24 h in 37 °C.

For Raman microspectroscopy, the bacteria were grown overnight (16–24 h) in 5 mL of Nutrient Broth media. The bacteria were then washed 3 times as described above. Finally, 10 μL of the washed bacterial cell suspension was put in droplets on Ni-foil discs and allowed to dry at room temperature for 15–60 min.

### Raman measurements

UVRR spectra were collected using a Raman microscope (HR800, Horiba/Jobin–Yvon) with a focal length of 800 mm. 244 nm of light was used for excitation, produced by a frequency-doubled line of an argon-ion laser (Coherent Innova 300, FReD). The laser was focused and directed through a × 40 antireflection-coated objective (LMU, NA: 0.5, UVB). Backscattered Raman light was collected through a 400-μm entrance slit into a 2400-lines/mm grating and detected by a nitrogen-cooled CCD camera. The spectral resolution was 2 cm^−1^. For each spectrum measured, 15 s of illumination time, and a maximal laser power of about 18 mW, was used leading to about 0.5 mW on the sample. During measurement, in order to avoid burning of the sample, the sample stage was rotated constantly in spiral manner. In each measurement, a time series of 10 spectra was obtained. A total of 25 time series were measured for each strain and replicate from 3 fused-silica slides. The spectra of each time series were averaged and considered as one spectrum to reduce noise. A total of ~ 750 spectra for each group (750 for the resistant group and 748 for the sensitive) were analyzed.

To measure individual bacterial cells using Raman microspectroscopy, a Raman microscope BioParticleExplorer (MicrobioID 0.5, RapID) was used. A 532-nm frequency-doubled solid-state Nd:YAG diode pumped laser (LCM-S-111, Laser-Export Company Ltd.) was used for excitation. The laser beam was focused with a × 100 magnification objective (MPLFLN × 100, NA: 0.9, Olympus Corporation) on the sample with a laser power of approximately 16 mW, leading to approximately 3.5 mW on the cells. Backscattered Raman light was focused to a single-stage monochromator (HE 532, Horiba Jobin Yvon) equipped with a 920-lines/mm grating and collected with a thermoelectrically cooled CCD camera (DV401A-BV, Andor Technology). The spectral resolution was approximately 10 cm^−1^. For each bacterial cell, two consecutive Raman spectra were measured at the same position, which were afterward combined. Integration time was 15 s for each bacterial cell. For each replicate, 60 spectra were collected. A total of > 2000 spectra for each group (2106 for the resistant group and 2062 for the sensitive) were analyzed.

### Statistical analysis

Prior to analysis, several preprocessing steps were conducted using the Ramanmetrix software [40]. Preprocessing included de-spiking the spectra (based on a method described before [41]), wavenumber calibration, background correction with a sensitive nonlinear iterative peak (SNIP) algorithm using 40 iterations, vector normalization, and cutting the spectra to the relevant range (500–1900 cm^−1^ for UVRR spectra and 400–3050 cm^−1^ for Raman microspectroscopy data). Also, for the Raman microspectroscopy data the silent region (1800–2800 cm^−1^) was removed.

Wavenumber calibration, with a polynomial fit function, was based on polystyrene spectra and 4-acetamidophenol spectra for UVRR and Raman microspectroscopy data, respectively. The polynomial degree was 2 for the UVRR spectra and 3 for the Raman microspectroscopy. A new reference spectrum was used for every measurement date.

Classification models were calculated individually for each dataset (UVRR and Raman microspectroscopy) using the Ramanmetrix software. We compared principal component analysis support vector machine (PCA-SVM) and principal component analysis linear discriminant analysis (PCA-LDA) models for both datasets and chose the optimal model and number of principal components based on the results of leave-one-strain-out cross-validation (LOSOCV). In this validation method, a model is trained repeatedly on the dataset excluding one strain, which is then predicted by the constructed model.

For the UVRR data, a PCA-SVM model was calculated, based on 4 PCs. For the Raman microspectroscopy data, a PCA-LDA model was calculated, based on 14 PCs.

For the Raman microspectroscopy data, prior to analysis, burned spectra were removed automatically using an in-house R script [42]. Furthermore, outliers were removed from the single-cell Raman spectra by using a correlation filter. In this method, the Pearson correlation coefficient with the average preprocessed spectrum of the entire dataset is calculated for each spectrum as described before [43]. The spectra with correlation values below 0.9 were excluded from the further analysis. More than 98% of spectra passed the filter.

After the classification models were calculated, we applied a “Majority Voting” approach. Because of in-sample heterogeneity, some spectra will classify incorrectly. In order to reach a decision for a strain (resistant/sensitive), a “vote” is conducted, and the class which has the majority of spectra is chosen as the “decided class.” This was accomplished using an in-house R script.

## Results and discussion

### Comparison of Raman spectra of resistant and sensitive bacteria

**Fig. 1:**
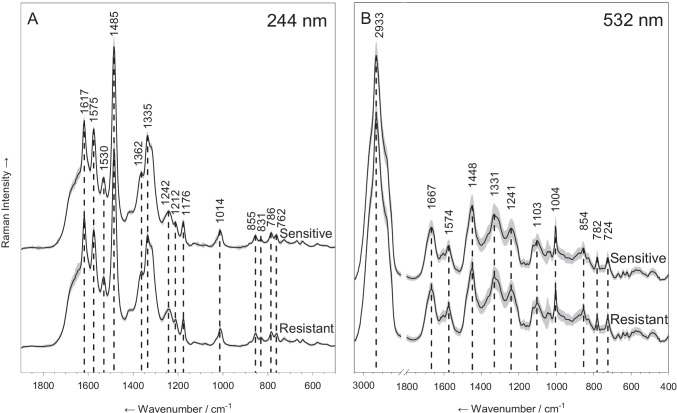
Mean spectra of resistant and sensitive *E. coli* strains. **A** Spectra measured with UVRR spectroscopy. **B** Spectra measured with Raman microspectroscopy. Standard deviations are shown as gray shades around each spectrum. All spectra are normalized and offset vertically for visualization

We measured the Raman spectra of 20 *E. coli* strains with two spectroscopic approaches: UV resonance Raman (UVRR) spectroscopy with excitation wavelength of 244 nm and Raman microspectroscopy with excitation wavelength of 532 nm. The mean spectra, comparing the resistant and sensitive strains are presented in Fig. 1. Figure 1A presents the results of UVRR spectroscopy and Fig. 1B the results of Raman microspectroscopy. The standard deviation of each mean spectrum is highlighted in gray.

The spectra measured by UVRR on bulk samples (Fig. 1A) represent primarily bands which are enhanced by the resonance effect of excitation with 244 nm. We selected this wavelength because of the resonance enhancement of the Raman signal. This enhancement occurs for molecules with absorption bands that are near the spectral region of the laser excitation. When excited with 244 nm light, the side chain vibrations of aromatic amino acids as well as the vibrations of the nucleobases of nucleic acids are enhanced. These represent two critical components of a bacterial cell: proteins and nucleic acids [28].

The spectrum exhibits a well-established pattern that is typical for bacterial spectra, where the peaks at 786, 1242, 1335, 1362, 1485, 1530, and 1575 cm^−1^ originate primarily from nucleic acid residues and the peaks at 762, 831, 855, 1014, 1176, 1212, and 1617 cm^−1^ from aromatic amino acids [28, 39, 44–48]. The peaks at 1242 and 1335 cm^−1^ represent a mixture of signals from nucleic acids and aromatic amino acids [44]. The exact band assignment can be seen in Table S3.

In Fig. 1B, the spectra collected from single cells with Raman microspectroscopy are presented. Here, as the samples were illuminated with 532 nm light, no strong resonance effect is seen. Also, the spectra exhibit a well-established pattern, typical for bacterial spectra, which represents a large array of biomolecules [11, 12, 14]. The peaks at 2933, 1448, and 854 cm^−1^ represent C-H stretching vibrations, CH_2_ deformation vibrations, and C–C/C–O–C stretching vibrations, respectively, which are abundant in many lipid, protein, and carbohydrate molecules. The peaks at 1667 and 1241 cm^−1^ are from amide I and amide III vibrations, respectively, and together with the sharp peak of the phenylalanine ring breathing vibration (1004 cm^−1^) represent proteins in the cell. The wide peak at 1331 cm^−1^ represents CH_2_ deformation vibrations in proteins and ring vibrations of guanine and adenine [49]. Lastly, weaker bands, such as 1574, 1103, 782, and 724 cm^−1^, represent nucleic acids [50, 51]. The full band assignment is presented in Table S4. Taken all together, these features present a larger, more phenotypic outlook on the chemistry of the cell than the one obtained by UVRR spectroscopy.

For both approaches, no clear difference can be discerned between the groups by observing the mean spectra. This finding is in accordance with previous works on the application of Raman spectroscopy on bacterial samples [11, 12, 14, 17, 28]. The chemical fingerprint of all bacterial species includes the same biomolecules (mostly nucleic acids, proteins, and lipids). As these differ slightly in proportions, structures, and composition, only slight differences exist between the spectra of different bacteria. The closer two strains are related, the smaller these differences are. This is especially evident here as all spectra examined come from the same species of bacteria, grown in the same conditions.

**Fig. 2:**
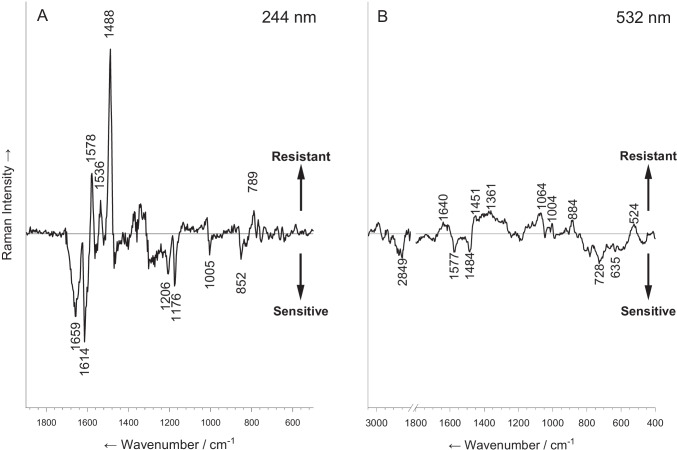
Difference spectra calculated by subtracting the mean spectrum of sensitive strains from the mean spectrum of resistant ones. **A** Spectrum derived from UVRR spectroscopy. **B** Spectrum derived from Raman microspectroscopy. The spectra are normalized and are presented in the same scale

However, slight differences can be found by multivariate statistical analysis and by calculating a difference spectrum. A difference spectrum was calculated by subtracting the mean spectrum of sensitive strains from that of resistant ones. In Fig. 2, difference spectra were calculated by subtracting the mean spectrum of sensitive strains from the mean spectrum of resistant ones: (A) spectrum derived from UVRR spectroscopy and (B) spectrum derived from Raman microspectroscopy. The spectra are normalized and are presented in the same scale. The difference spectrum is presented for both UVRR and Raman microspectroscopy, and the peak assignments are detailed in Fig. 2. For the UVRR spectrum, as detailed in Table 1, the Raman bands which have a stronger signal in resistant bacteria are 1578 and 1536 (guanine), 1488 (nucleic acid marker), and 789 cm^−1^ (cytosine) [39, 52, 53]. All these vibrations represent nucleic acid residues. In contrast, the Raman signals which are enhanced for sensitive strains were 1659, 1614, 1206, 1176, 1005, and 852 cm^−1^. These peaks represent the aromatic amino acids phenylalanine, tyrosine, tryptophan, and histidine, which are indicators for proteins [28]. These findings suggest that in resistant bacteria, we see an increase in nucleic acid/protein ratio.

**Table 1:** Assignment of Raman bands in the difference spectrum calculated from UVRR spectroscopy. The original annotations as described in the literature are given in brackets. Sensitive: signals with larger peaks for sensitive strains (negative values); resistant: signals with larger peaks for resistant strains (positive values)

Wavenumber/cm^−1^		Assignment (wavenumber/cm^−1^)	Biomolecular group
Sensitive	Resistant		
1659		T, C, U, Phe, amide I (1650–1655) [39]	DNA/RNA, protein
1614		Tyr, Trp, Phe (1615) [39]	Protein
1206		Tyr (1209) [28]	Protein
1176		His (1171) [39, 48]	Protein
1005		Phe (1004) [53]	Protein
852		Tyr (851) [45]	Protein
	1578	G, Trp [46]	DNA/RNA, protein
	1536	G (1535–1543) [54]	DNA/RNA
	789	C, U (782) [39]	DNA/RNA

Abbreviations: *T* thymine, *C* cytosine, *U* uracil, *Phe* phenylalanine, *Tyr* tyrosine, *Trp* tryptophan, *His* histidine, *G* guanine

For the Raman microspectroscopy data, where no resonance effect is present, bands of all biomolecules are present in both the resistant and sensitive isolates. The assignments are given in Table 2. Raman bands which have a stronger signal in resistant bacteria include nucleic acids, represented by cytosine and thymine bands at 1640 and 1361 cm^−1^ and proteins, represented by bands 1064 and 1004 cm^−1^ (C–C/C-N stretching vibrations and the ring breathing of phenylalanine, respectively). Lastly, the bands assigned to lipids are at 884 cm^−1^ (C-O–O skeletal vibrations) and 1451 cm^−1^ (CH_2_ deformation vibrations). Whereas for sensitive isolates, the nucleic acids are represented by vibrations at 728, 1484, and 1577 cm^−1^ (guanine, adenine), proteins at 635 (tryosine skeletal vibrations), and lastly lipids by the band at 2849 cm^−1^ (for CH_2_ stretching vibrations). These features show again the presence of a phenotypic outlook of the bacterial biochemical composition that is detected by the 532 nm excitation. However, the difference spectrum consists of broad, superimposed Raman signals with low intensity. Although there are peaks identifiable, they are not as prominent as in UVRR and are therefore difficult to interpret and negligible (Fig. 2B).

**Table 2:** Assignment of Raman bands in the difference spectrum calculated from Raman microspectroscopy. The original annotations as described in the literature are given in brackets. Sensitive: signals with larger peaks for sensitive strains (negative values); resistant: signals with larger peaks for resistant strains (positive values)

Wavenumber/cm^−1^		Assignment (wavenumber/cm^−1^)	Biomolecular group
Sensitive	Resistant		
1640		C (1640) [28]	DNA/RNA
1451		δ(CH_2_) (1440–1460) [50, 51]	Protein, lipid
1361		C, T (1369) [39, 45]	DNA/RNA
1064		ν(C––C), ν(C––N) (1061) [50]	Protein
1004		Phe (1004) [46]	Protein
884		C-O–O (866–898) [55]	Lipid
524		ν(S–S) (520–540), δ(C–O–C) glycosidic ring (540)[39, 55]	Protein, carbohydrate
	2849	ν(CH_2_) (2832–2862) [56]	Lipid
	1577	G, A (1575–1578) [50, 51]	DNA/RNA
	1484	G, A (1480) [28, 46]	DNA/RNA
	728	A (730) [51, 53]	DNA/RNA
	635	Tyr [50, 51]	Protein

Abbreviations: δ = deformation vibrations, ν = stretching vibrations, *T* thymine, *C* cytosine, *U* uracil, *Phe* phenylalanine, *Tyr* tyrosine, *Trp* tryptophan, *His* histidine, *G* guanine

The findings from the UVRR difference spectrum can be explained by the fact that multi-resistant strains carry, beside their core genome, resistance genes. These genes are present on plasmids, bacterial chromosomes, and mobile genetic elements. When translated, they allow the bacteria to overcome the toxic effect of antibiotics in their environment. Resistance genes, which developed with the extensive use of antibiotics over the past 80 years, are part of an evolutionary process in the genome of multi-resistant strains [57]. These genes affect the composition of DNA in the cell both qualitatively and quantitatively: as resistant strains contain more genetic material compared to sensitive ones. This change in the genetic composition is likely the cause of the differences seen with UVRR spectroscopy.

In the 532 nm excitation, no resonance enhancement of the nucleic acids occurs, and thus, the small changes in nucleic acid content and composition are not observed. From a biochemical perspective, the overall metabolism of these different strains does not appear to have changed significantly. This is not surprising, since the bacteria were not exposed to antibiotics. Resistance mechanisms are often not expressed unless the cell is under stress and therefore the metabolism and phenotype remained nearly identical.

Recently, a similar Raman setup was used to study the difference between sensitive strains of *E. coli* and strains which were engineered to be resistant. One study simulated the acquisition of a resistance plasmid in a native *E. coli* strain by molecular engineering, and measured the bacteria with Raman microspectroscopy and UV resonance Raman spectroscopy [39]. Another study used Raman spectroscopy to discriminate between lab-evolved resistant *E. coli* strains [58]. Both studies found that the differentiation between the resistant and sensitive strains was indeed related to the nucleic acid/protein ratios. Moreover, in both studies, the ratio was larger for resistant strains. This supports our similar findings in the present study on clinical, naturally occurring bacteria.

### Classification of resistant and sensitive strains

In order to assess the ability of Raman spectroscopy to differentiate between resistant and sensitive strains, a machine learning classification model was trained. Two common classification models, SVM for UVRR and LDA for Raman microspectroscopy, were used to analyze the differences in the spectra. The model parameters, such as number of principal components, were optimized using the leave-one-strain-out cross-validation (LOSOCV) method. The classification results are presented in Table 3. In each table, Raman spectra were classified into their respective class. The accuracy, sensitivity, and specificity of each model are also presented. For both UVRR data and Raman microspectroscopy data, the models performed with 60% accuracy. This indicates that the differentiation of resistant and sensitive strains is possible, though limited, with both methods by applying machine learning algorithms.

**Table 3:** Summary of the classification models results for Raman spectra in confusion matrices for UVRR and Raman microspectroscopy with 532 nm excitation. The true labels are shown by row and the predicted classes by column. Correctly identified spectra are shown in bold

UVRR spectroscopy		Prediction		Accuracy/%	Sensitivity/%	Specificity/%
		Resistant	Sensitive			
Reference	Resistant	524	226	60.1	58.5	62.5
	Sensitive	371	377		62.5	58.5
Raman microspectroscopy		Prediction		Accuracy/%	Sensitivity/%	Specificity/%
		Resistant	Sensitive			
Reference	Resistant	1250	833	59.5	59.4	59.6
	Sensitive	856	1229		59.6	59.4

This finding is surprising given that the difference spectra of the two methods differed significantly. However, this antagonism can be explained by the dimension reduction and manipulation of the data by the machine learning method. These methods highlight slight differences and revalues common features. Thus, even very minor differences, which are not observable in the spectra can be of significance, as demonstrated before [11, 59].

### Application of majority voting to improve classification

When measuring bacteria with Raman spectroscopy, many spectra are measured for each isolate. Since not all the bacteria are perfectly identical, a natural heterogeneity can be seen in the spectra. Especially in the single cell measurements the heterogeneity is larger due to cell to cell variations originating from differences in growth stage and metabolism. In bulk measurements, heterogeneity is not as intense, since each spectrum is composed of signals from thousands of cells. For clinical decision-making, there is a need for precise results, with concrete information about the resistance characteristics. Therefore, this heterogeneity must be resolved, providing a read-out that does not refer to individual spectra but to an entire bacterial strain.

To eliminate this problem, we applied a majority voting system on the confusion matrix of the classification models. In this system, the sum of decisions is calculated and a vote is taken, where for each strain, a majority of classifications decides whether the strain is classified as resistant or sensitive. The result of majority voting per strain is shown in Table 4. For the UVRR spectroscopy data, the accuracy of the model improved from 60% in the original model to 70% after voting, reaching a correct classification of 14/20 strains. For the Raman microspectroscopy data, we observed an increase in accuracy, from 60 to 75%, correctly classifying 15/20 strains.

**Table 4:** Summary of the classification models’ results after majority voting in confusion matrices for UVRR and Raman microspectroscopy with 532 nm excitation. The true labels are shown by row and the predicted classes by column. Correctly identified spectra are shown in bold

UVRR spectroscopy		Prediction		Accuracy/%	Sensitivity/%	Specificity/%
		Resistant	Sensitive			
References	Resistant	9	1	70	90	50
	Sensitive	5	5		50	90
Raman microspectroscopy		Prediction		Accuracy/%	Sensitivity/%	Specificity/%
		Resistant	Sensitive			
Reference	Resistant	7	2	75	70	80
	Sensitive	3	8		80	70

This means that overall accuracy, with majority voting, has increased by 10–15%. For the UVRR data, the sensitivity of the model improves much more, from 60 to 90% (meaning 9/10 resistant strains were correctly identified). Furthermore, the problem of sample heterogeneity has been mediated; as for each strain, only one decision remains. The increase in accuracy, together with removal of heterogeneity, helps define the presence of resistance in the strains and is useful for clinical decision-making.

The misclassified strains are summarized in Table S5. Interestingly, only the sensitive strain DSM 498 is misclassified by both methods. The other nine misclassified strains are unique.

Overall, we found that UVRR spectroscopy and Raman microspectroscopy provide similar classification results. This finding is surprising because we expected the UVRR to significantly outperform Raman microspectroscopy due to the stronger S/R ratio and the differences in the spectra (Fig. 2). The ability to differentiate resistance from UVRR was mostly dependent on resonance-enhanced signals. For single-cell spectra excited with 532 nm light, this resonance enhancement does not occur. It is likely that for these spectra, the classification is related not only to changes in genetic content, but also to the larger metabolic changes which a resistant cell undergoes. Resistant bacteria show a decline in overall fitness even when grown in optimal conditions. This decline is related to a loss in enzyme efficiency, changes in cell wall thickness, and membrane porin content. While each of these changes is important in resistance, they will not affect the spectra dramatically. They are slight changes in the overall metabolic profile of a cell [60–62]. However, it seems that even such slight changes are present in the spectra, and by using a machine learning algorithm, they enabled classification as efficient as that achieved with UVRR spectroscopy. This finding is the most important finding of this study. It highlights the applicability of both approaches in detecting microbial susceptibility and can guide future studies in the flexibility of Raman-based diagnostics. With machine learning algorithms, one can extract information from nonresonant spectra that is as useful as resonance-enhanced spectra—for a clinical application.

It is important to note that since different wavelengths were employed as excitation sources, as well as different sample preparation methods (bulk and single cells), a direct comparison of the performances of UVRR spectroscopy and Raman microspectroscopy cannot be made. The same exact cell was never measured twice, and the same exact sample was not prepared for both devices. However, this is not the focus of this study. This study focuses on presenting the ability of different approaches using Raman spectroscopy and analyzing their potential individual diagnostic value.

One may argue about whether this method is suitable for clinical laboratory settings since the accuracy provided is only fair. However, the improvement of overall accuracy and sensitivity by majority voting evidenced that by removing the natural heterogeneity within the strain, the presence of resistance can be revealed. Since the number of stains used in this study was small, it is interesting to see how the diagnostic potential is shown. Further studies are required, with larger bacterial cohorts and a larger biodiversity of strains and species in order create a database suitable for hospital settings. The biggest advantage of Raman spectroscopy compared to traditional microbiology methods is its high speed in providing results. By providing treating physicians with information on bacterial resistance early, many unnecessary prescriptions of last-line antibiotics can be avoided. This could allow better management of hospital resistance rates, and hinder the rise of antimicrobial resistance.

## Conclusions

In the present study for the first time, ESBL and CRE clinical *E. coli* isolates could be distinguished from sensitive strains using Raman spectroscopy. We show that UVRR spectra provide better S/R ratio and a change in nucleic acid/protein ratio can be observed. Yet, in contrast to what was expected, when using machine learning algorithms to classify the data, both methods were comparable. This comparison was done for the first time on clinically relevant data and future studies should consider these methods comparable to some extent. Lastly, we found majority voting is key to minimizing the natural heterogeneity and make Raman spectroscopy more suitable for clinical application.

This study presents another facet of Raman spectroscopy–based diagnostics. We conclude this method could enable better, rapid, label-free antibiotic resistance diagnostics in clinical settings. For future studies, especially those with clinical applications, we recommend applying a majority voting approach to clarify results into actionable information. Further work is still essential to improve classification for use in healthcare. The classification accuracy found in the study is limited for application, but likely with a larger and more diverse dataset, it could improve, providing better diagnostics to help fight the rise of antimicrobial resistance and for the improvement of global health.

## Supplementary Information

Below is the link to the electronic supplementary material.

Supplementary file1 (PDF 249 KB)

## Data Availability

The data presented in this study are available on request from the corresponding author.
