# Supplementary material for: Detection of multi-resistant clinical strains of E. coli with Raman spectroscopy
Source: Anal Bioanal Chem. 2022 Jan 4;414(4):1481–92. doi: 10.1007/s00216-021-03800-y (PMC8761712; doi:10.1007/s00216-021-03800-y)
Supplement: Supplementary file 1 — Supplementary file1 (PDF 249 KB) [file 216_2021_3800_MOESM1_ESM.pdf]

Supplementary Material

Detection of multi-resistant clinical strains of *E. coli* with Raman Spectroscopy

Amir Nakar[1,2,3*], Aikaterini Pistiki[1,2,3*], Oleg Ryabchykov[1,2*], Thomas Bocklitz[1,2,3], Petra Rösch[2,3] and Jürgen Popp[1,2,3,4]

1. Leibniz Institute of Photonic Technology Jena (a member of Leibniz Health Technologies), Albert-Einstein-Straße 9, 07745 Jena, Germany
2. Institute of Physical Chemistry and Abbe Center of Photonics, Friedrich Schiller University, Helmholtzweg 4, 07743 Jena, Germany
3. Research Campus Infectognostics e.v. Jena, Germany, Philosophenweg 7, 07743, Jena, Germany
4. Jena Biophotonics and Imaging Laboratory, Albert-Einstein-Straße 9, 07745 Jena, Germany

* Equal Contribution

Table S1 List of strains used in the study, their resistance class and source

Strain	Resistance	Source
Gr2	ESBL / Resistant	Gr
Gr5	ESBL / Resistant	Gr
Gr6	ESBL / Resistant	Gr
Gr7	ESBL / Resistant	Gr
Gr9	ESBL / Resistant	Gr
PAK56	CRE / Resistant	PAK
PAK57	CRE / Resistant	PAK
PAK58	CRE / Resistant	PAK
PAK64	CRE / Resistant	PAK
PAK66	CRE / Resistant	PAK
ATCC25922	Sensitive	ATCC
DSM9031	Sensitive	DSMZ
DSM3871	Sensitive	DSMZ
DSM1576	Sensitive	DSMZ
DSM8696	Sensitive	DSMZ
DSM429	Sensitive	DSMZ
DSM498	Sensitive	DSMZ
DSM499	Sensitive	DSMZ
DSM501	Sensitive	DSMZ
DSM613	Sensitive	DSMZ

Gr: Strains isolates from patient samples in the University of Thessaly hospital, Greece.
PAK: Strains collected in a survey of CREs in a Pakistani Hospital (ref Braun SD, et al. Future Microbiol. 2018)
DSMZ: German Collection of Microorganisms and Cell Cultures GmbH
ATCC: American Type Culture Collection

Table S2 Antibiotic susceptibility to extended spectrum β-lactam and carbapenem antibiotics

Evaluated using a VITEK 2 Compact (bioMérieux) with VITEK® 2 AST cards (bioMérieux). The test was run according to the manufacturer's instructions.

Sensitive
Intermediate
Resistant
Not Tested

		Aztreonam	Cefepime	Cefotaxime	Ceftazidime	Ciprofloxacin	Imipenem [CRE]	Meropenem [CRE]	Moxifloxacin	Piperacillin	Piperacillin/ Tazobactam	Tigecycline	Tobramycin	Trimethoprim/ Sulfamethoxazole	Fosfomycin	Colisin
Gr02	ESBL	Intermediate	Intermediate	Resistant	Intermediate	Resistant	Sensitive	Sensitive	Resistant	Resistant	Resistant	Sensitive	Resistant	Resistant	Not Tested	Not Tested
Gr05	ESBL	Resistant	Resistant	Resistant	Resistant	Resistant	Sensitive	Sensitive	Resistant	Resistant	Sensitive	Sensitive	Resistant	Sensitive	Not Tested	Not Tested
Gr06	ESBL	Resistant	Intermediate	Resistant	Intermediate	Sensitive	Sensitive	Sensitive	Sensitive	Resistant	Sensitive	Sensitive	Resistant	Resistant	Not Tested	Not Tested
Gr07	ESBL	Not Tested	Intermediate	Not Tested	Resistant	Resistant	Sensitive	Sensitive	Not Tested	Not Tested	Sensitive	Not Tested	Not Tested	Resistant	Not Tested	Not Tested
Gr09	ESBL	Resistant	Intermediate	Resistant	Resistant	Resistant	Sensitive	Sensitive	Resistant	Resistant	Resistant	Sensitive	Resistant	Sensitive	Not Tested	Not Tested
PAK56	CRE	Resistant	Intermediate	Resistant	Resistant	Resistant	Resistant	Resistant	Resistant	Resistant	Resistant	Sensitive	Resistant	Sensitive	Sensitive	Sensitive
PAK57	CRE	Resistant	Resistant	Resistant	Resistant	Resistant	Intermediate	Resistant	Resistant	Resistant	Resistant	Sensitive	Sensitive	Resistant	Sensitive	Sensitive
PAK58	CRE	Resistant	Resistant	Resistant	Resistant	Resistant	Resistant	Resistant	Resistant	Resistant	Resistant	Sensitive	Resistant	Resistant	Sensitive	Sensitive
PAK64	CRE	Intermediate	Resistant	Resistant	Resistant	Resistant	Resistant	Resistant	Resistant	Resistant	Resistant	Sensitive	Sensitive	Sensitive	Sensitive	Sensitive
PAK66	CRE	Resistant	Resistant	Resistant	Resistant	Resistant	Resistant	Intermediate	Resistant	Resistant	Resistant	Sensitive	Resistant	Resistant	Sensitive	Sensitive
ATCC25922	Sensitive	Sensitive	Sensitive	Sensitive	Sensitive	Sensitive	Sensitive	Sensitive	Sensitive	Sensitive	Sensitive	Sensitive	Sensitive	Sensitive	Sensitive	Sensitive
DSM9031	Sensitive	Sensitive	Sensitive	Sensitive	Sensitive	Sensitive	Sensitive	Sensitive	Sensitive	Sensitive	Sensitive	Sensitive	Sensitive	Sensitive	Sensitive	Sensitive
DSM3871	Sensitive	Sensitive	Sensitive	Sensitive	Sensitive	Sensitive	Sensitive	Sensitive	Sensitive	Sensitive	Sensitive	Sensitive	Sensitive	Sensitive	Sensitive	Sensitive
DSM6969	Sensitive	Sensitive	Sensitive	Sensitive	Sensitive	Sensitive	Sensitive	Sensitive	Sensitive	Sensitive	Sensitive	Sensitive	Sensitive	Sensitive	Sensitive	Sensitive
DSM8696	Sensitive	Sensitive	Sensitive	Sensitive	Sensitive	Sensitive	Sensitive	Sensitive	Sensitive	Sensitive	Sensitive	Sensitive	Sensitive	Sensitive	Sensitive	Sensitive
DSM429	Non Pathogenic	Sensitive	Sensitive	Sensitive	Sensitive	Sensitive	Sensitive	Sensitive	Sensitive	Sensitive	Sensitive	Sensitive	Sensitive	Sensitive	Sensitive	Sensitive
DSM498	Non Pathogenic	Sensitive	Sensitive	Sensitive	Sensitive	Sensitive	Sensitive	Sensitive	Sensitive	Sensitive	Sensitive	Sensitive	Sensitive	Sensitive	Sensitive	Sensitive
DSM499	Non Pathogenic	Sensitive	Sensitive	Sensitive	Sensitive	Sensitive	Sensitive	Sensitive	Sensitive	Sensitive	Sensitive	Sensitive	Sensitive	Sensitive	Sensitive	Sensitive
DSM501	Non Pathogenic	Sensitive	Sensitive	Sensitive	Sensitive	Sensitive	Sensitive	Sensitive	Sensitive	Sensitive	Sensitive	Sensitive	Sensitive	Sensitive	Sensitive	Sensitive
DSM613	Non Pathogenic	Sensitive	Sensitive	Sensitive	Sensitive	Sensitive	Sensitive	Sensitive	Sensitive	Sensitive	Sensitive	Sensitive	Sensitive	Sensitive	Sensitive	Sensitive

Table S3 Raman band assignments for the mean spectra collected with UV Resonance Raman Spectroscopy

**Wavenumber (cm^{-1})**	**Assignment [references]**	**Biochemical Group**
762	Trp (760) [13]	Protein
786	C, U, T (780-786) [4, 5]	Nucleic acids
831	Tyr (829) [14]	DNA/RNA
855	Tyr (854) [4, 12]	Protein
1014	Trp, Phe ring breathing (1006-1009) [1, 3, 4]	Protein
1176	C-C and C-N stretching vibration of Cytosine and Thymine (1175), Tyr(1175) [6, 12]	Proteins, Nucleic acids
1212	Phe, Tyr (1209) [4, 12]	Protein
1242	T (1237) [11]	Nucleic acids
1335	G, A (1337) [5]	Nucleic acids
1362	C, T (1369) [6, 8, 9]	Nucleic acids
1485	G, A (1480) [6]	Nucleic acids
1530	C (1527-1529) [1, 7]	Nucleic acids
1575	G, A (1578) [9,13]	Nucleic acids
1617	Tyr, Trp (1619) [6,12]	Protein

* Abbreviations: A: adenine, T: thymine, C: cytosine, G: guanine, U: uracil, Trp: tryptophan, Tyr: tyrosine, Phe: phenylalanine.

Table S4 Raman band assignments for the mean spectra collected with Raman microspectroscopy

**Wavenumber (cm^{-1})**	**Assignment [references]**	**Biochemical Group**
725	T (752) [8, 9]	DNA
782	C (783) [9, 10]	DNA/RNA
854	CC / COC str (853) [9, 10]	All protein, lipids and carbohydrates
1004	Phe (1004) [3, 4]	Protein
1103	>PO2− str (1090) [9, 10]	DNA
1241	Amide III (1220–1290) [11]	Protein
1331	C–H_2 def (in proteins), G, A (1334)[12]	DNA / Protein
1448	C–H_2 def (1440–1460) [9, 10]	All protein, lipids and carbohydrates
1574	G, A (1575–1578) [9, 10]	DNA
1667	Amide I (1650–1680) [9, 10]	Protein
2933	C–H str (2935) [2, 9]	All protein, lipids and carbohydrates

* Abbreviations: A: adenine, T: thymine, C: cytosine, G: guanine, U: uracil, Trp: tryptophan, Tyr: tyrosine, Phe: phenylalanine, His: histidine, str: stretching mode, def: deformation mode

Table S5. List of misclassified strains after majority voting. The class of the bacteria is given in brackets

**Misclassified UVRR**	**Misclassified Raman Microspectroscopy**
**DSM498 (Sensitive)**	**DSM498 (Sensitive)**
DSM429 (Sensitive)	DSM501 (Sensitive)
DSM499 (Sensitive)	Gr09 (ESBL)
DSM613 (Sensitive)	Pak58 (CRE)
DSM1576 (Sensitive)	Pak64 (CRE)
Gr02 (ESBL)	

References

1. Walter A, Reinicke M, Bocklitz T, Schumacher W, Rösch P, Kothe E, et al. Raman spectroscopic detection of physiology changes in plasmid-bearing Escherichia coli with and without antibiotic treatment. Analytical and Bioanalytical Chemistry. 2011;400(9):2763-73.
2. Harz M, Claus RA, Bockmeyer CL, Baum M, Rösch P, Kentouche K, et al. UV-resonance Raman spectroscopic study of human plasma of healthy donors and patients with thrombotic microangiopathy. Biopolymers. 2006;82(4):317-24.
3. De Gelder J, De Gussem K, Vandenabeele P, Moens L. Reference database of Raman spectra of biological molecules. Journal of Raman Spectroscopy. 2007;38(9):1133-47.
4. Töpfer N, Müller MM, Dahms M, Ramoji A, Popp J, Slevogt H, et al. Raman spectroscopy reveals LPS-induced changes of biomolecular composition in monocytic THP-1 cells in a label-free manner. Integr Biol (Camb). 2019.
5. Žukovskaja O, Kloß S, Blango MG, Ryabchykov O, Kniemeyer O, Brakhage AA, et al. UV-Raman Spectroscopic Identification of Fungal Spores Important for Respiratory Diseases. Anal Chem. 2018;90(15):8912-8.
6. Silge A, Heinke R, Bocklitz T, Wiegand C, Hipler U-C, Rösch P, et al. The application of UV resonance Raman spectroscopy for the differentiation of clinically relevant Candida species. Analytical and Bioanalytical Chemistry. 2018;410(23):5839-47.
7. Tu AT. Raman Spectroscopy in Biology : Principles and Applications. New York: Wiley; 1982.
8. Uzunbajakava N, Lenferink A, Kraan Y, Willekens B, Vrensen G, Greve J, et al. Nonresonant Raman imaging of protein distribution in single human cells. Biopolymers. 2003;72(1):1-9.
9. Huang WE, Li M, Jarvis RM, Goodacre R, Banwart SA. Chapter 5 - Shining Light on the Microbial World: The Application of Raman Microspectroscopy. Advances in applied microbiology. 70: Academic Press; 2010. p. 153-86.
10. Maquelin K, Kirschner C, Choo-Smith LP, van den Braak N, Endtz HP, Naumann D, et al. Identification of medically relevant microorganisms by vibrational spectroscopy. J Microbiol Methods. 2002;51(3):255-71.
11. Schuster KC, Urlaub E, Gapes JR. Single-cell analysis of bacteria by Raman microscopy: spectral information on the chemical composition of cells and on the heterogeneity in a culture. J Microbiol Methods. 2000;42(1):29-38.

12. Kloß S, Kampe B, Sachse S, Rösch P, Straube E, Pfister W, et al. Culture Independent Raman Spectroscopic Identification of Urinary Tract Infection Pathogens: A Proof of Principle Study. Analytical Chemistry. 2013;85(20):9610-6.
13 Harz M, Krause M, Bartels T, Cramer K, Rösch P, Popp J. Minimal Invasive Gender Determination of Birds by Means of UV-Resonance Raman Spectroscopy. Analytical Chemistry. 2008;80(4):1080-6.
14. Czamara K, Majzner K, Pacia MZ, Kochan K, Kaczor A, Baranska M. Raman spectroscopy of lipids: a review. Journal of Raman Spectroscopy. 2015;46(1):4-20.
15. Azemtsop Matanfack G, Taubert M, Guo S, Bocklitz T, Küsel K, Rösch P, et al. Monitoring Deuterium Uptake in Single Bacterial Cells via Two-Dimensional Raman Correlation Spectroscopy. Analytical Chemistry. 2021;93(21):7714-23.
